# Defining double burden of malnutrition across individual, household and population level: A narrative review

**DOI:** 10.1111/1747-0080.70037

**Published:** 2025-08-08

**Authors:** Ashis Talukder, Matthew Kelly, Md Abu Sayeed, Darren Gray, Haribondhu Sarma

**Affiliations:** ^1^ National Centre for Epidemiology and Population Health Australian National University Canberra Australian Capital Territory Australia; ^2^ Statistics Discipline, Science Engineering and Technology School Khulna University Khulna Bangladesh; ^3^ Population Health QIMR Berghofer Medical Research Institute Brisbane Queensland Australia

**Keywords:** definition of DBM, double burden of malnutrition, level of DBM assessment, systematic narrative review

## Abstract

**Aims:**

This study aims to evaluate the existing definitions of double burden of malnutrition and to synthesise and propose operational definitions at different levels, with the goal of improving consistency in measurement and policy development.

**Methods:**

We conducted a narrative review that used structured search and narrative synthesis to review and summarise how double burden of malnutrition has been defined at different levels. We performed a structured search across PubMed, Web of Science and Scopus, focusing on peer‐reviewed articles published between January 2010 and December 2024. We screened articles for relevance to double burden of malnutrition and categorised them based on the level of the definition, such as households, individuals and populations.

**Results:**

Of the 60 selected studies, the majority originated from Latin America (*n* = 19, 32%), followed by South Asia (*n* = 10, 17%) and Southeast Asia (*n* = 8, 13%). At the household level, the most common definition of double burden of malnutrition was the coexistence of an overweight/obese mother and a stunted child. Individual‐level definitions commonly included the coexistence of overweight/obesity with anaemia or stunting. However, definitions at the population level varied considerably and lacked consistency across studies.

**Conclusions:**

Our findings highlight the need for consistent operational definitions of double burden of malnutrition across different levels of analysis. Drawing on existing literature, we synthesise practical definitions at different levels to more accurately reflect the coexistence of different forms of malnutrition. Clarifying these definitions can improve the comparability of prevalence estimates across contexts and support the development of more effective, evidence‐based strategies to address the growing burden of double burden of malnutrition.

## 1 INTRODUCTION

The double burden of malnutrition refers to the simultaneous presence of undernutrition and overnutrition within households, individuals or populations.^1^, ^2^, ^3^, ^4^, ^5^ This concept was first formally introduced at the 1992 International Conference on Nutrition, where it was defined primarily at the national level as the coexistence of overweight or obesity alongside undernutrition within a country.^6^ Since then, significant shifts in dietary patterns, physical activity, disease burdens and life expectancy have transformed the global nutritional landscape, especially in many low‐ and middle‐income countries.^7^, ^8^

As a result, the double burden of malnutrition is now increasingly recognised not only as a national‐level issue but also as a phenomenon occurring at more localised levels—within households and even individuals.^1^, ^9^ It remains a critical public health challenge, as highlighted in major research and publications, including *The Lancet* series titled *The Double Burden of Malnutrition*, published in December 2019.^1^, ^10^, ^11^, ^12^ The double burden of malnutrition undermines progress towards global health goals, exacerbates health inequalities and places dual pressure on healthcare systems by contributing to both infectious diseases and non‐communicable diseases (NCDs).^11^, ^12^ This evolving nutritional reality—characterised by persistent undernutrition (e.g., stunting, wasting and micronutrient deficiencies) alongside a growing prevalence of overweight, obesity and diet‐related NCDs—poses significant challenges to achieving sustainable development and advancing health equity in low‐ and middle‐income countries.^9^, ^13^, ^14^, ^15^

Globalisation and urbanisation are exacerbating this nutritional burden by creating environments where processed foods high in fats, sugars and salts increasingly replace traditional diets rich in whole grains, fruits and vegetables.^13^ This shift has increased the double burden prevalence, especially in low‐ and middle‐income countries, where undernutrition persists in children and overnutrition occurs in adults living in the same households.^1^, ^16^, ^17^ In these contexts, the coexistence of undernutrition and overnutrition within the same individual is also becoming more common, as children who are undernourished in early childhood are more likely to become overweight in adulthood, heightening their risk for NCDs.^11^ These overlapping challenges demand integrated interventions that address both aspects of malnutrition.

Despite growing recognition of the double burden of malnutrition, a key research gap lies in the absence of specific operational definitions and indicators, which hampers the development, implementation and evaluation of effective nutrition‐related initiatives.^17^ While the World Health Organization (WHO) outlines a broad conceptual framework,^9^ it does not provide detailed operational guidance for defining and measuring double burden of malnutrition across individual, household and population levels. Early studies typically defined it as the presence of an overweight or obese mother and a stunted child within the same household,^18^, ^19^, ^20^ but recent research has expanded this to include a wider range of anthropometric and biochemical outcomes.^21^ This heterogeneity in operational definitions and measurement criteria not only results in inconsistent estimates of the prevalence of double burden of malnutrition but also hampers trend monitoring, cross‐study comparability and the formulation of coordinated public health strategies.^22^, ^23^ Although a previous review identified existing operational definitions of the double burden of malnutrition and their frequency of use,^17^ it neither proposed specific definitions tailored to individual, household and population levels nor attempted to construct a universal framework requiring global consensus. This gap may limit the ability to assess the full scope of the problem and develop consistent, evidence‐based responses.

It is important to note that the term ‘triple burden of malnutrition’ has recently gained attention in research and practice to describe the simultaneous presence of undernutrition, micronutrient deficiencies and overnutrition.^24^, ^25^, ^26^ The triple burden of malnutrition builds on the idea of the double burden by specifically including micronutrient deficiencies as a separate issue,^27^, ^28^ broadening the conceptualisation of malnutrition challenges. Moreover, micronutrient deficiencies can accompany either over‐ or under‐nutrition in different ways and therefore this paper will focus on the most clearcut concept. Accordingly, this review focuses on the double burden definitions and indicators, where ongoing inconsistencies continue to hinder effective policy and programme development.^17^

In response to this challenge, this paper aims to review the definitions and indicators of the double burden of malnutrition used in current literature so far, with the goal of identifying gaps in the operationalisation of this concept. By exploring the different levels of double burden malnutrition—individuals, households and populations—and analysing the indicators used to assess malnutrition at each of these levels, this study seeks to provide a clearer framework for future research. This paper also aims to propose specific operational definitions of the double burden of malnutrition at different levels, along with practical measurement criteria that are essential for improving the accuracy of prevalence estimates and for guiding the development and monitoring of effective nutrition policies that address both undernutrition and overnutrition.

## 2 METHODS

We performed a narrative review guided by systematic search strategies. We adopted a structured, transparent and rigorous approach, including clearly defined inclusion and exclusion criteria, PRISMA reporting and independent screening by two reviewers, with disagreements resolved by consensus involving a third reviewer if necessary. We did not register a protocol, conduct a formal risk of bias assessment or perform meta‐analysis, as the heterogeneity of definitions and conceptual approaches made quantitative synthesis inappropriate. This hybrid approach aligns with the methodology of structured narrative reviews described previously,^29^, ^30^ and the review process is reported in accordance with the checklist PRISMA 2020 statement.^31^ As the primary aim was to synthesise existing operational definitions rather than to conduct a quantitative synthesis, we did not register a formal systematic review protocol.

To aid readers from diverse disciplines, we provided detailed explanations of key technical terms used in this paper, such as operational definition of the double burden of malnutrition, systematic narrative review, stunting, wasting, obesity and diet‐related NCDs in Table 1.

**TABLE 1:** Explanation of key terminologies used in this study.

Key terminologies	Explanation
Operational definition of DBM	A specific and measurable definition used to identify or assess a concept in research or practice. In this study, it refers to how DBM is conceptually described in the literature and measured or assessed in applied research.
Systematic narrative review	A method of reviewing literature that follows a structured process but presents the findings through a narrative synthesis.
Stunting^a^	Stunting means a child is shorter than expected for their age. It usually happens when they do not get enough nutritious food for a long time or suffer from frequent illnesses. Stunting is often linked to poverty, poor health and lack of proper care in early childhood. It can affect both a child's growth and brain development.
Wasting^a^	Wasting means a child is too thin for their height. It usually happens because of not getting enough food or from losing weight due to illnesses like diarrhoea. Wasting can be serious and increase the risk of death in young children, but it can be treated with the right care and nutrition.
Overweight and obesity^a^	Overweight and obesity occur when a person has too much body fat for their height, which can harm their health. These conditions happen when a person takes in more energy (kilojoules or calories) than they burn through activity. Globally, people are eating more high‐sugar and high‐fat foods and are less physically active, which contributes to this problem.
Diet‐related non‐communicable diseases (NCDs)^a^	Diet‐related NCDs include heart disease, stroke, certain cancers and diabetes. They are mainly caused by unhealthy eating habits and poor nutrition, which are major causes of illness around the world.

Abbreviations: DBM, double burden of malnutrition; NCDs, non‐communicable diseases.

^a^ *Source*: World Health Organization.^32^

We conducted a structured search for peer‐reviewed journal articles to ensure methodological transparency and quality. This search focused on articles published between January 2010 and December 2024. This timeframe was selected to capture the most recent developments in the conceptualisation and measurement of the double burden of malnutrition. For instance, the WHO Global Nutrition Policy Review, based on a 2009–2010 survey, highlighted growing policy interest in integrated approaches to undernutrition, micronutrient deficiencies and overnutrition—the key components of the double burden of malnutrition framework.^33^

In our search strategy, we used related terms, such as ‘double burden of malnutrition,’ ‘dual burden of malnutrition,’ ‘stunted child or overweight mother’ and acronyms (such as DBM, SCOWT and SCOM). The acronym ‘SCOWT’ (stunted child and overweight mother) has been used in previous literature to define the double burden of malnutrition.^34^ A detailed overview of the search strategy is provided in Supporting Information S1.

To meet the objectives of our review, we included those studies published in scientific peer‐reviewed journals based on the following criteria: (1) studies that provide clear definitions or conceptual frameworks of the double burden of malnutrition at the individual, household or population level; (2) analyses assessing the overlap between ‘overnutrition’ and ‘undernutrition’; (3) studies that focus on households where one member is undernourished and another is overweight; (4) studies focused on individuals experiencing both undernutrition and overnutrition at the same time (e.g., overweight individual who is suffering from anaemia, etc); (5) studies aiming to address the double burden of malnutrition at the country level.

The exclusion criteria for this review are: (1) studies that focused exclusively on either undernutrition or overweight/obesity, without addressing their coexistence; (2) review articles; (3) research dealing solely with the clinical or biochemical aspects of malnutrition, without any link to the double burden of malnutrition; (4) studies that examined nutrition interventions but did not define or conceptualise the double burden of malnutrition; (5) grey literature; (6) we limited our review to English‐language publications.

Furthermore, we included studies that explicitly defined and operationalised the double burden of malnutrition—either by formulating their own definitions or by explicitly applying a recognised definition, such as those from WHO. Studies that only mentioned the double burden of malnutrition without specifying how it was defined or measured were excluded. This ensured that the review focused on studies that contributed clear and applicable definitions, though we acknowledge this may have excluded some studies reflecting heterogeneity in practice.

We conducted a comprehensive literature search using three major electronic databases: PubMed, Scopus and Web of Science. The initial search yielded 2675 articles in total—868 from PubMed, 936 from Scopus and 871 from Web of Science. These records were imported into EndNote™ X9.2, where duplicates were identified and removed. After removing duplicate articles, we retained 1204 unique articles for the title and abstract screening phase.

Two reviewers independently screened the titles and abstracts of the 1204 unique articles using pre‐defined inclusion and exclusion criteria. This initial screening identified 258 articles as potentially relevant, which were then selected for full‐text review. At this stage, both reviewers were blinded to each other's selections to minimise bias. The inter‐rater agreement during this phase was substantial, with a Cohen's *κ* of 0.84, indicating a high level of consistency between reviewers. Discrepancies in selection were discussed to reach a consensus, and a third reviewer was consulted when necessary to resolve disagreements.

**FIGURE 1:**
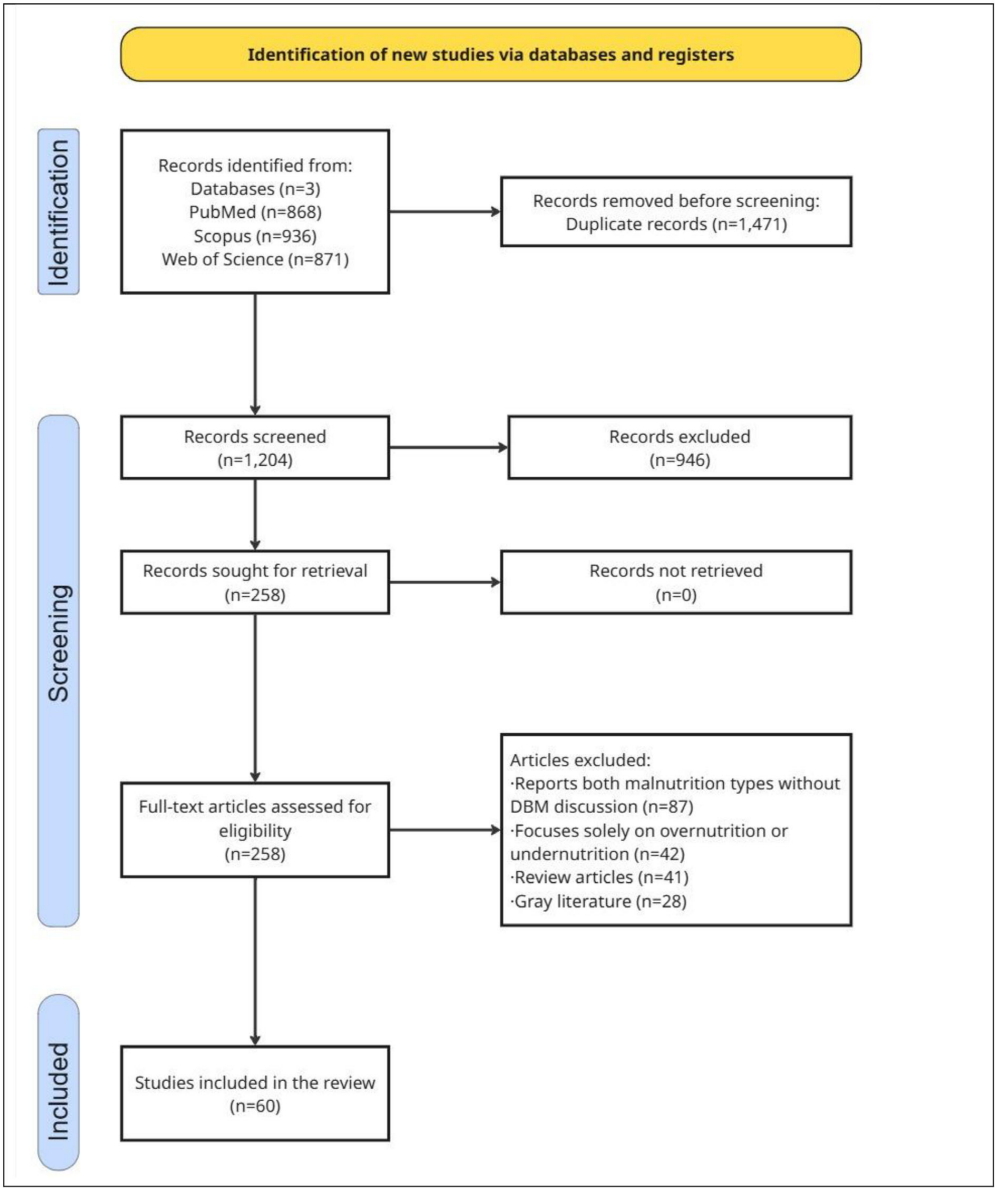
Flow diagram illustrating the identification, screening, eligibility assessment and inclusion of studies for the review. DBM, double burden of malnutrition.

During the full‐text screening phase, the same pre‐defined inclusion and exclusion criteria were applied as in the abstract screening stage. Each of the 258 articles was read in full by both reviewers, and detailed notes were maintained on the reasons for exclusion. The inter‐rater agreement at this stage was also strong, with a Cohen's *κ* of 0.81, reflecting consistent application of the criteria. As before, any differences in judgement were resolved through discussion, with the involvement of the third reviewer if consensus could not be achieved. Following this thorough review, 198 articles were excluded for not meeting the eligibility criteria. As a result, 60 articles were included in the final analysis. The complete selection process is illustrated in Figure 1.

For each of the 60 articles included in the final review, we used a structured data extraction process to collect detailed information relevant to the review objectives. The extracted data focused on three core domains: (a) how the double burden of malnutrition was defined in each study; (b) the anthropometric or nutritional indicators used to measure undernutrition and overnutrition; and (c) the cut‐off points or thresholds applied to classify nutritional status. Additional contextual information, such as year of publication, country or region of study and the level of analysis (individuals, households or populations), was also recorded (see Table S1).

We used a narrative synthesis approach to examine how the double burden of malnutrition has been conceptualised and measured across the included studies. Full‐text articles were imported into NVivo 12, where a coding framework was developed to identify both the level of assessment (individual, household or population) and the relevant definitions. Text segments containing definitions were coded under a dedicated node, allowing for systematic extraction and organisation of definitions by assessment level. In addition, each article was coded to identify the presence of key terms such as ‘double burden,’ ‘dual burden,’ ‘co‐occurrence,’ ‘co‐existence’ and ‘double and triple burden’ within the text segments defining the double burden of malnutrition. Frequency counts for these terms were generated using NVivo's text query functions. All coding was independently verified by a second reviewer to ensure consistency. The coded data were exported into Microsoft Excel (2016) to facilitate tabulation and preparation for figure generation in R (version 4.4.0). Based on this synthesis, we proposed a set of operational definitions at each level of assessment, incorporating practical and feasible cut‐off points to enhance consistency and comparability in future research.

This review article is based on previously published studies and does not involve any new studies with human participants or animals conducted by the authors. As such, ethical approval and informed consent were not required.

## 3 RESULTS

**FIGURE 2:**
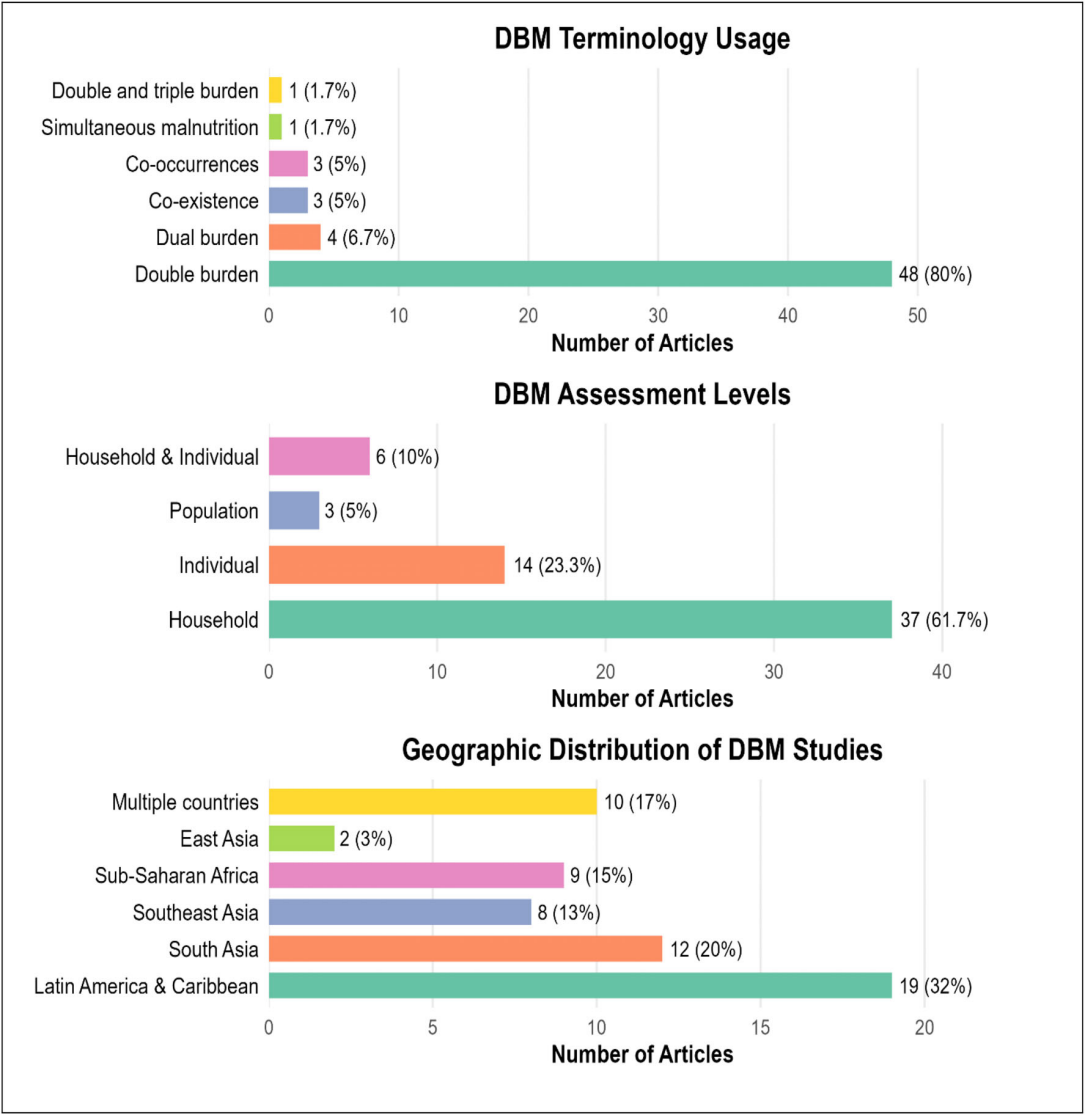
Key dimensions and research contexts based on geographical setting of double burden of malnutrition (DBM) reported across 60 included studies. The usage of DBM‐related terminology was assessed by reviewing the definitions of included articles to identify which specific terms each article used to describe the double burden of malnutrition. For example, 48 articles used the term ‘double burden’ and four articles used ‘dual burden’ when defining or identifying DBM.

Of the 60 included articles, the majority (62%, *n* = 37) focused on the household level, followed by 23% (*n* = 14) at the individual level and 5% (*n* = 3) at the population level. Additionally, 10% (*n* = 6) of studies examined both household and individual levels. The term ‘double burden’ appeared most frequently in literature (80% of articles, *n* = 48) followed by ‘dual burden’ (6.7%, n = 4). Other related terms such as ‘co‐occurrence,’ ‘co‐existence’ and ‘double and triple burden’ appeared too rarely to be reported separately. In terms of geographical distribution, Latin America and the Caribbean accounted for the highest proportion of studies (32%, *n* = 19), followed by South Asia (20%, *n* = 12), Southeast Asia (13%, *n* = 8) and Sub‐Saharan Africa (15%, *n* = 9). A smaller number of studies were conducted in East Asia (3%, *n* = 2). Furthermore, 17% (*n* = 10) of the studies included data from multiple countries (Figure 2).

Table 2 shows how the double burden of malnutrition has been operationally defined at different levels of measurement (households, individuals and populations) and shows the frequency of each specific definition across the 60 included studies. At the household level, a total of 62 coded definitions were identified. The most common definition—used in 25 studies (40.3%)—was the coexistence of an overweight or obese mother and a stunted child. A broader definition, pairing an overweight or obese mother with any form of child malnutrition (stunting, wasting or underweight), appeared in 19 studies (30.6%). Less frequent definitions included households with an overweight or obese mother and a wasted child (three studies, 4.8%), an overweight/obese mother and a child with micronutrient deficiency (two studies, 3.2%) or an anaemic child (five studies, 8.1%). Combinations such as an overweight child with an underweight mother, an overweight/obese mother with an underweight child or an overweight child with an anaemic mother were each reported in two studies (3.2%). Rare definitions included adult overweight paired with child overweight or adult underweight paired with child underweight (one study each, 1.6%).

**TABLE 2:** Summary of available operational definitions of double burden of malnutrition by assessment level.

Level of measurement	Operational definition	Total number of occurrences *n* (%)^a^
Household level	Overweight/obese mother and stunted child	25 (40.3)
	2 Overweight/obese mother and child having any form of malnutrition (stunting, wasting and underweight)	19 (30.6)
	3 Overweight/obese mother and wasted child	3 (4.8)
	4 Overweight/obese mother and child facing micronutrient deficiency	2 (3.2)
	5 Overweight/obese mother and anaemic child	5 (8.1)
	6 Overweight child and underweight mother	2 (3.2)
	7 Overweight/obese mother and underweight child	2 (3.2)
	8 Overweight child and anaemic mother	2 (3.2)
	9 Adult overweight and child overweight	1 (1.6)
	10 Adult underweight and child underweight	1 (1.6)
Individual level	Micronutrient deficiency and at least one cardiometabolic risk factor	2 (6.3)
	2 Underweight and at least one cardimetabolic risk factor	1 (3.1)
	3 Overweight/obesity and micronutrient deficiency within an individual/children	9 (28.1)
	4 Overweight/obesity and anaemia within an individual/children	9 (28.1)
	5 Overweight and stunting among under five children	8 (25.0)
	6 Anaemia and at least one cardiometabolic risk factor	1 (3.1)
	7 Underweight and anaemia within an individual/children	2 (6.3)
Population level	DBM measured by stunting and overweight thresholds: if (stunting >30% and overweight in children or adults >20%) or (wasting >15% and overweight in children or adults >20%) or (thinness in women >20% and overweight in children or adults >20%)	1 (33.3)
	2 DBM in a country when adult overweight and underweight prevalence >10%	1 (33.3)
	3 DBM is measured by the prevalence of stunting and overweight, using thresholds of 20% for stunting and 10% for overweight	1 (33.3)

*Note*: Micronutrient deficiency includes vitamin A deficiency or iodin deficiency or vitamin D deficiency or any combination of these indicators.

Abbreviation: DBM, double burden of malnutrition

^a^ Many articles included multiple DBM definitions. In the table, the total number of occurrences of specific DBM definitions across the 60 included articles is 97; percentages are calculated based on occurrences within each assessment level: household (*n* = 62), individual (*n* = 32) and population (*n* = 3).

At the individual level, 32 coded definitions were identified. The most frequently used definitions—each reported in nine studies (28.1%)—were: (a) coexistence of overweight or obesity and a micronutrient deficiency (e.g., vitamin A, iodine or vitamin D); and (b) coexistence of overweight or obesity and anaemia within the same individual. The combination of overweight and stunting among children under five was used in eight studies (25.0%). Less common definitions included micronutrient deficiency combined with at least one cardiometabolic risk factor (two studies, 6.3%), underweight combined with a cardiometabolic risk factor (one study, 3.1%), anaemia combined with a cardiometabolic risk factor (one study, 3.1%) and the coexistence of underweight and anaemia (two studies, 6.3%).

At the population level, three distinct definitions were identified, each used in a single study (33.3%). Although the limited number of studies restricts generalisability, these examples reflect considerable variability in how the double burden of malnutrition is defined at this level. All three definitions were based on the simultaneous high prevalence of undernutrition and overweight/obesity within a population. One study applied thresholds such as stunting >30% and overweight >20%, or wasting >15% and overweight >20%, or underweight in women >20% and overweight >20%. Another defined the double burden of malnutrition as the coexistence of adult overweight and underweight, each exceeding 10% within the same year. The third used a combination of stunting >20% and overweight >10%. This variation in indicator selection and threshold values highlights the need for clearer, evidence‐based definitions to improve consistency and comparability across future studies.

## 4 DISCUSSION

The diverse definitions of the double burden of malnutrition identified in our review point to deeper conceptual and methodological challenges in establishing a unified definition. This variability is driven by the complexity of malnutrition, shifting nutrition patterns in low‐ and middle‐income countries and inconsistent data availability.^1^, ^35^, ^36^ Since the double burden of malnutrition typically encompasses undernutrition, micronutrient deficiencies and overweight/obesity, which may coexist at the individual, household or population levels,^1^, ^2^, ^3^ these overlapping conditions make standardisation particularly challenging. Researchers often adapt definitions to fit specific study goals, populations or datasets, leading to inconsistent indicators, cut‐offs and measurement approaches.^37^, ^38^, ^39^, ^40^ The absence of universally accepted criteria from global bodies further widens this gap. While some degree of heterogeneity is methodologically justifiable, it limits comparability across studies and complicates the design of coherent public health responses.^17^ This review revealed considerable heterogeneity in how the double burden of malnutrition is conceptualised and defined across studies. At the individual level, definitions often combined overweight with undernutrition within the same individual, though specific indicators and cut‐offs varied. At the household level, most studies defined it narrowly as an overweight mother and undernourished child dyad, ignoring other intra‐household combinations. At the population level, definitions varied in the indicators and thresholds used, with some deviating from WHO standards.

These variations reflect differences in theoretical frameworks, data availability and cultural contexts, which complicate comparability. Recognising these gaps, we proposed operational definitions that address the observed inconsistencies. Specifically, our definitions expand the household‐level conceptualisation beyond the mother–child dyad, align individual‐ and population‐level indicators with WHO benchmarks and ensure measurability using commonly available data sources such as Demographic and Health Surveys and Multiple Indicator Cluster Surveys. This approach bridges the conceptual and methodological gaps identified in the literature.

We found that household‐level studies most defined the double burden of malnutrition as the coexistence of an overweight or obese mother and a stunted child.^17^, ^19^, ^20^, ^34^, ^35^, ^41^, ^42^, ^43^, ^44^, ^45^, ^46^, ^47^, ^48^, ^49^, ^50^, ^51^, ^52^, ^53^, ^54^, ^55^, ^56^ While this definition is practical—largely due to the availability of maternal and child data—it overlooks other important within‐household nutritional disparities. Several studies called for broader definitions to include other dyads (e.g., underweight mothers with overweight children, or intergenerational combinations involving elderly members).^57^, ^58^, ^59^, ^60^, ^61^, ^62^, ^63^, ^64^, ^65^, ^66^, ^67^, ^68^, ^69^, ^70^, ^71^, ^72^, ^73^, ^74^ Building on these insights, our proposed household‐level definition encompasses a wider range of within‐household nutritional imbalances, making it more inclusive and context‐sensitive.

At the individual level, we found a wide range of definitions.^37^, ^38^, ^39^, ^40^, ^75^, ^76^, ^77^, ^78^, ^79^, ^80^, ^81^, ^82^, ^83^, ^84^, ^85^, ^86^, ^87^ Most commonly, the double burden of malnutrition is defined as the coexistence of overweight, obesity and anaemia in the same individual.^37^, ^38^, ^39^, ^40^, ^49^, ^75^, ^76^, ^77^ Some studies also examine combinations involving overweight or obesity with micronutrient deficiencies or stunting.^77^, ^78^, ^79^, ^80^, ^81^, ^82^, ^83^ A few have extended the definition further to include cardiometabolic risk factors such as diabetes and hypertension.^76^, ^84^ Although these risk factors are important for understanding overall health, they reflect the consequences or risks of chronic diseases,^88^ rather than the core nutritional imbalances central to the the double burden of malnutrition concept. To preserve conceptual clarity, it is important to distinguish it from the broader double burden of disease. In line with the previous literature, we argue that the double burden of malnutrition should primarily focus on the coexistence of undernutrition and overnutrition,^1^, ^2^, ^3^, ^4^, ^5^ particularly in nutritional terms, rather than encompassing broader health risks. Including cardiometabolic risk factors may mix up nutrition problems with broader health issues that require different policy solutions. However, we acknowledge that the topic remains an area of ongoing debate, and future research may wish to explore hybrid definitions, especially in ageing or urban populations where such overlaps are more prevalent.^89^, ^90^

At the population level, definitions often vary due to the use of different prevalence thresholds for undernutrition and overweight or obesity. For instance, one study defines the double burden of malnutrition based on a stunting prevalence of 20% and an overweight prevalence of 10%,^87^ while another uses thresholds such as 10% or more for both adult underweight and overweight in a year.^91^ These inconsistencies in threshold selection lead to varying estimates prevalence, making cross‐country comparisons and trend analyses difficult. As a result, monitoring global progress and evaluating policy effectiveness become challenging. Previous research has highlighted that the absence of standardised thresholds can result in underestimating or overestimating the burden of the double burden of malnutrition,^92^ potentially misguiding resource allocation and public health responses. In light of these issues, one study recommended establishing globally consistent criteria based on empirical evidence of associated health risks.^92^ Our proposed threshold values aim to reflect this balance between empirical relevance and feasibility, but we also acknowledge the need for future validation in diverse country settings.

In our review, most studies relied on anthropometric indicators—such as stunting, wasting, underweight and overweight—to define and measure the double burden of malnutrition. While these indicators are widely used and offer useful insights, researchers always need to take special care so that the choice aligns with the specific aims of a study.^17^ For instance, studies focused on maternal and child health commonly use the combination of maternal BMI and child stunting,^35^, ^45^ whereas research on adult population may require measures such as anaemia status and thinness or overweight.^38^, ^70^ This variation highlights that definitions and the selection of indicators should be informed by the research context and clearly aligned with study objectives.^17^, ^93^ Choosing indicators that match the population, setting and purpose of the study enhances the accuracy assessment and ensures that the resulting findings are more relevant and effective in addressing both the prevalence and underlying causes of the double burden of malnutrition in different settings. On the other hand, misalignment between indicators and study objectives can lead to inaccurate assessments and misleading conclusions. For example, while BMI is a widely used measure, it does not reflect body fat distribution and can underestimate obesity‐related health risks in certain demographic groups.^94^

While our review highlights the importance of consistent definitions, it is equally important to recognise that its manifestation and measurement are shaped by environmental and contextual factors. Socioeconomic conditions, urbanisation, food systems, healthcare access and cultural practices all influence how different forms of malnutrition coexist.^95^, ^96^, ^97^, ^98^, ^99^ For instance, rapid urbanisation often leads to greater consumption of energy‐dense, nutrient‐poor foods and sedentary lifestyles, contributing to overweight and obesity.^95^, ^96^, ^97^ In contrast, rural areas may continue to struggle with food insecurity and undernutrition.^98^, ^99^ Therefore, adopting a definition that is grounded in its core concept but adapted to the study's specific objectives can enhance the relevance of research findings and increase the effectiveness of existing policies across diverse settings.

Given the existing challenges in defining the double burden of malnutrition using consistent nutrition indicators, we propose operational definitions at the individual, household and population levels. These definitions are derived from our synthesis of how it has been conceptualised across the included studies, and they are informed by both empirical patterns in the literature and international guidance to ensure consistency with global public health standards. Importantly, these definitions are designed to be both conceptually robust and practically measurable. Specifically, they can be operationalised using widely available data sources such as Demographic and Health Surveys, Multiple Indicator Cluster Surveys and other routine surveillance systems that collect anthropometric and micronutrient data at the individual and household levels. This approach bridges conceptual and methodological gaps and improves comparability of findings across contexts.

The specific operational definitions and corresponding cut‐off points for each nutrition indicator are described below and further explained in Table 3, along with the justification for each selected threshold. Researchers can use these definitions based on the aim of the research. Proposed definitions at different levels are:

**TABLE 3:** Cut‐off points for measuring under and overnutrition with their rationale.

Level of measurement	Category	Cut‐off points	Reason for considering the cut‐off^a^
Household and Individual level DBM	Anthropometric measurements		
	Undernutrition		
	Stunting	• HAZ <−2 SD	Global standard cut‐off proposed by WHO^100^
	Wasting	• WHZ <−2 SD	
	Underweight	• WAZ <−2 SD • BAZ <− 2 SD	
	Underweight for adult	• BMI <18.5 kg/m^2^	
	Overnutrition		
	Overweight for adults	• BMI >25 kg/m^2^	
	Overweight for under five children	• WHZ >+2 SD • BAZ >+2 SD	
	Overweight for adults (Asian population)	• BMI >23 kg/m^2^	Due to the increased risk of cardiovascular disease and diabetes at lower BMI levels among Asians compared to non‐Asians, the WHO recommended in 2004 using a modified threshold of 23.0 kg/m^2^ to define overweight for Asians instead of 25.0 kg/m^2^ ^101^
	Biochemical measurements		
	Anaemia	• Haemoglobin concentrations <12.0 g/dL (female) • Haemoglobin concentrations <13.0 g/dL (Male)	Worldwide accepted cut‐off proposed by WHO.^102^
	Vitamin A deficiency	• Serum retinol levels, with a cut‐off of <10 μg/dL	Worldwide accepted cut‐off proposed by WHO.^103^
	Iodine insufficiency	• A urinary iodine excretion (UIE) of <50 μg/dL indicate iodine insufficiency	Previous studies used this cut‐off.^104^
	Vitamin D deficiency	• Based on the guidelines of the American Society of Endocrinology, vitamin D deficiency can be characterised by Serum 25‐hydroxyvitamin D [25(OH)D] concentration levels below 20 ng/mL.	Previous studies used this cut‐off.^105^
Population level DBM	Population level	• For adults: prevalence of both over and undernutrition is greater than 10% • For children under five: Stunting prevalence >20%; Wasting prevalence >10%; Overweight prevalence >10%	For adult: A 10% prevalence was selected as a threshold for public health concern, as WHO and previous studies in LMICs indicate that adult underweight (BMI <18.5 kg/m^2^) or overweight at this level signals poor nutrition and warrants public health attention.^92^ For children under 5 years of age, a national prevalence of stunting above 20% or wasting above 10% and a prevalence of overweight above 10% has been suggested as indicating public health concerns.^106^

Abbreviations: BAZ, body‐mass‐index‐for‐age *Z* scores; BMI, body‐mass‐index; DBM, double burden of malnutrition; HAZ, height for age *Z* score; LMIC, low‐ and middle‐income countries; SD, standard deviation; WHZ, weight for height *Z* score.

^a^ The cut‐off points summarised in the table were selected based on established global standards and widely accepted benchmarks, primarily drawing from World Health Organization (WHO) guidelines and commonly cited thresholds in nutrition literature. While some referenced reports and studies were not part of the primary pool of articles included in this review, they were incorporated to provide authoritative and practical guidance for defining malnutrition indicators relevant to DBM.

Household level: At the household level, the double burden of malnutrition occurs when there is a coexistence of contrasting forms of malnutrition within the same household. This can manifest as an adult member being overweight or obese while a child is undernourished, or an adult member being underweight while a child is overnourished, or when two household members—adults or children—experience opposite forms of malnutrition, with one being overweight and the other undernourished.

Individual level: At the individual level, the double burden of malnutrition is defined as the simultaneous presence of overweight or obesity alongside stunting, anaemia or other micronutrient deficiencies in the same individual, occurring at the same time during a given developmental stage (e.g., childhood, adolescence or adulthood).

Population level: At the population level, the double burden of malnutrition refers to the coexistence of a high prevalence of undernutrition and overweight or obesity within the same population. It is identified when the prevalence of overweight or obesity in children or adults exceeds 10%, alongside a stunting prevalence of over 20% or a wasting prevalence of over 10% or a underweight prevalence of over 10%.

This study has several advantages. By systematically examining definitions and indicators across individual, household and population levels, this study provides a comprehensive framework that supports more targeted and effective interventions. Our rigorous methodology, which includes well‐defined inclusion and exclusion criteria as well as independent screening by multiple reviewers, enhances the reliability of our analysis and minimises selection bias. Additionally, all data extraction was cross‐checked by a second reviewer to ensure consistency and accuracy. However, this study also has limitations. We did not register a protocol or conduct formal risk‐of‐bias assessments. Excluding grey literature from the systematic search may have omitted relevant evidence, although authoritative grey literature (e.g., WHO guidelines) was cited to justify our proposed thresholds. The heterogeneity of study designs (e.g., cross‐sectional vs. longitudinal) and our focus on explicit definitions may have excluded studies reflecting real‐world diversity.

In our research, we propose practical definitions of the double burden of malnutrition at the household, individual and population levels that are more feasible for measurement. These proposed definitions may inform researchers and policymakers in the development of more effective strategies to address its rising prevalence. We also emphasise that clear and specific definitions are critical for designing robust research methodologies and formulating effective policy initiatives. Importantly, using consistent definitions can help incorporate the double burden of malnutrition indicators more effectively into national health surveillance systems and global monitoring frameworks, such as the Sustainable Development Goals and the WHO Global Nutrition Targets. Moreover, by carefully selecting indicators that align with the specific objectives of the research—whether for prevalence estimation, trend analysis or intervention evaluation—we can ensure those findings are both relevant and impactful.

## AUTHOR CONTRIBUTIONS

AT and HS conceptualised and designed the study. AT and MAS conducted the literature search, while HS resolved any discrepancies in the selected articles. AT led the data analysis and interpretation of the results. AT drafted the manuscript, with HS, MK and DG supervising the analysis output. HS, MK, MAS and DG critically revised the manuscript for intellectual content. All authors reviewed and edited subsequent drafts, read and approved the final version and contributed to the decision to submit for publication.

## CONFLICT OF INTEREST STATEMENT

No potential competing interest was reported by the author(s).

## Supporting information


**Data S1.** Supporting Information.

## Data Availability

Data sharing is not applicable to this article as no new data were created or analyzed in this study.
