# Supplementary material for: Defining double burden of malnutrition across individual, household and population level: A narrative review
Source: Nutr Diet. 2025 Aug 8;83(1):8–22. doi: 10.1111/1747-0080.70037 (PMC12884263; doi:10.1111/1747-0080.70037)
Supplement: Supplementary file 1 — Data S1. Supporting Information. [file NDI-83-8-s001.docx]

**Supplementary documents**

**Search Strategy**

**PubMed**

((("double burden" OR "dual burden" OR simultaneous OR co-occurrence OR co-existing OR concurrent ) OR malnutrition OR DBM OR SCOWT OR SCOM ) AND (stunt* OR undernutrition OR underweight OR thinness OR wasting OR malnutrition )) AND (individual* OR house* OR population OR "overweight mother*" OR "underweight child*" OR "overweight child*" )

**Scopus**

((("double burden" OR "dual burden" OR simultaneous OR co-occurrence OR co-existing OR concurrent ) OR malnutrition OR DBM OR SCOWT OR SCOM ) AND (stunt* OR undernutrition OR underweight OR thinness OR wasting OR malnutrition )) AND (individual* OR house* OR population OR "overweight mother*" OR "underweight child*" OR "overweight child*" )

**Web of Science**

((("double burden" OR "dual burden" OR simultaneous OR co-occurrence OR co-existing OR concurrent ) OR malnutrition OR DBM OR SCOWT OR SCOM ) AND (stunt* OR undernutrition OR underweight OR thinness OR wasting OR malnutrition )) AND (individual* OR house* OR population OR "overweight mother*" OR "underweight child*" OR "overweight child*" )

**Supplementary table 1: Summary table of the review**

| **No.** | **Title** | **Reference ID** | **Level of measurement** | **Total participants** | **Region** | **Indicators with cut off** | **Definition of DBM** |
| --- | --- | --- | --- | --- | --- | --- | --- |
|  | Double Burden of Malnutrition and Its Associated Factors in Urbanized Indigenous Peoples (Orang Asli) of Peninsular Malaysia | Shariff et al. (2024) | Household | 451 households | Malaysia (specifically, the state of Selangor) | - Stunting: <-2SD height-for-age - Wasting: <-2SD weight-for-height - Underweight: <-2SD weight-for-age - Overweight/obese: BMI ≥25 kg/m² | DBM is defined as overweight mother and child having any form of undernutrition (stunting, wasting or underweight). |
|  | Prevalence and socio-demographic factors associated with double and triple burden of malnutrition among mother-child pairs in India: Findings from a nationally representative survey (NFHS-5) | Ramasubramani *et al.* (2024) | Household | 167,380 mother-child pairs | India | - Stunting: <-2SD height-for-age - Wasting: <-2SD weight-for-height - Underweight: <-2SD weight-for-age - Overweight/obese: BMI ≥25 kg/m² | DBM is defined as overweight mother and child having any form of undernutrition (stunting, wasting or underweight). |
|  | Magnitude, trends and drivers of the coexistence of maternal overweight/obesity and childhood undernutrition in Ethiopia: Evidence from Demographic and Health Surveys (2005-2016) | Pradeilles *et al.* (2024) | Household | 13,107 households | Ethiopia | - Stunting: <-2SD height-for-age - Anemia: hemoglobin level <11.0 g/dl | - Household-level DBM: coexistence of maternal overweight/obesity and child undernutrition  - Type 1: Maternal overweight/obesity and under-five stunting  - Type 2: Maternal overweight/obesity and under-five anemia  - Type 3: Maternal overweight/obesity and under-five anemia and/or stunting |
|  | Dynamics of the double burden of malnutrition in Guatemala: a secondary data analysis of the demographic and health surveys from 1998-2015 | Sagastume et al. (2024) | Household | Total: 39,749 households | Guatemala | - Stunting: <-2SD height-for-age - Wasting: <-2SD weight-for-height - Underweight: <-2SD weight-for-age   Overweight/obese: BMI ≥25 kg/m² | DBM is defined as overweight mother and child having any form of undernutrition (stunting, wasting or underweight). |
|  | Comparison of household double burden of malnutrition among mother-child dyads in different settings in Maharashtra | Jeyakumar et al. (2024) | Household | Total: 295 mother-child dyads | Maharashtra, India | - Stunting: <-2SD height-for-age - Wasting: <-2SD weight-for-height - Underweight: <-2SD weight-for-age - Overweight/obese: BMI ≥25 kg/m² | DBM is defined as overweight mother and child having any form of undernutrition (stunting, wasting or underweight). |
|  | Double burden of malnutrition and associated factors among mother-child pairs at household level in Bahir Dar City, Northwest Ethiopia: community based cross-sectional study design | Mekonnen et al. (2024) | Household | Total: 702 mother-child pairs (initially), with 661 participating in the study. | Ethiopia | Undernutrition for children:   - Stunting: <-2SD height-for-age - Wasting: <-2SD weight-for-height - Underweight: <-2SD weight-for-age   Overweight for children   - Overweight: >+2SD height-for-age   Overnutrition for adults:   - Overweight/obese: BMI ≥25 kg/m²   Undernutrition for adults:   - Underweight: BMI <18.5 | Participants were categorised as experiencing DBM if either the mother was overweight/obese and the child was undernourished (with stunting, wasting, or underweight), or if the mother was underweight and the child was overweight |
|  | Socioeconomic determinants of the double burden of malnutrition among women of reproductive age in sub-Saharan Africa: A cross-sectional study | Okyera et al. (2024) | Household | Total: 97,529 women | sub-Saharan Africa | - Stunting: <-2SD height-for-age   Overweight/obese: BMI ≥25 kg/m² | DBM in households, defined as the coexistence of an overweight/obese mother and a stunted child |
|  | Association Between Parental Education and Simultaneous Malnutrition Among Parents and Children in 45 Low-and Middle-Income Countries | Chen et al. (2023) | Household | Total: 480,060  - Mother-child pairs: 423,340  - Father-child pairs: 56,720 | multinational (45 LMICs for mother-child pairs and 16 LMICs for father-child pairs) | Undernutrition for children:   - Stunting: <-2SD height-for-age - Wasting: <-2SD weight-for-height - Underweight: <-2SD weight-for-age - Anemia: hemoglobin level <11.0 g/dl   Overweight for children   - Overweight: >+2SD height-for-age   Overnutrition for adults:   - Overweight/obese: BMI ≥25 kg/m²   Undernutrition for adults:   - Underweight: BMI <18.5 - Short stature (mother’s height <145 cm; father’s height <155 cm)   Anemia: hemoglobin level <12 g/dL | DBM is classified into four subtypes: (1) maternal (or paternal) overnutrition with child undernutrition, (2) maternal (or paternal) undernutrition with child overnutrition, (3) maternal (or paternal) overnutrition with child overnutrition, and (4) maternal (or paternal) undernutrition with child undernutrition. |
|  | Association between maternal stature and household-level double burden of malnutrition: findings from a comprehensive analysis of Ethiopian Demographic and Health Survey | Sahiledengle et al. (2023) | Household | 33,454 mother-child pairs | Ethiopia | - Stunting: <-2SD height-for-age - Wasting: <-2SD weight-for-height - Underweight: <-2SD weight-for-age - Overweight/obese: BMI ≥25 kg/m² | DBM is defined as overweight mother and child having any form of undernutrition (stunting, wasting or underweight). |
|  | Malnutrition in mother-child dyads in the Brazilian National Survey on Child Nutrition (ENANI-2019) | Farias et al. (2023) | Household | 13,659 mother-child dyads | Brazil | - Stunting: <-2SD height-for-age - Wasting: <-2SD weight-for-height - Underweight: <-2SD weight-for-age - Overweight/obese: BMI ≥25 kg/m² | DBM is defined as overweight mother and child having any form of undernutrition (stunting, wasting or underweight). |
|  | Global inequalities in the double burden of malnutrition and associations with globalisation: a multilevel analysis of Demographic and Health Surveys from 55 low-income and middle-income countries, 1992-2018 | Seferidi et al. (2022) | Household | 1,132,069 child-mother pairs | Multinational (55 low-income and middle-income countries) | - Stunting: <-2SD height-for-age - Overweight/obese: BMI ≥25 kg/m² | DBM is defined as a stunted child with an overweight mother living in the same household |
|  | Drivers and distribution of the household-level double burden of malnutrition in Bangladesh: analysis of mother-child dyads from a national household survey | Sarker et al. (2022) | Household | Total: 8697 mothers-child pair | Bangladesh | Undernutrition for children:   - Stunting: <-2SD height-for-age - Wasting: <-2SD weight-for-height - Underweight: <-2SD weight-for-age   Overweight for children   - Overweight: >+2SD height-for-age   Overnutrition for adults:   - Overweight/obese: BMI ≥25 kg/m²   Undernutrition for adults:   - Underweight: BMI <18.5 | DBM at the household level, defined as:  - Coexistence of mothers' underweight condition and children's overweight condition  - Coexistence of mothers' overweight and children's stunting, wasting, or underweight condition |
|  | Patterns and Determinants of Double Burden Malnutrition at Household Level in Babylon | Zahraa et al. (2022) | Households | 205 households | Iraq, Babylon governorate | - Stunting: <-2SD height-for-age - Wasting: <-2SD weight-for-height - Underweight: <-2SD weight-for-age - Overweight/obese: BMI ≥25 kg/m² | DBM is defined as overweight mother and child having any form of undernutrition (stunting, wasting or underweight). |
|  | Transition and persistence in the double burden of malnutrition and overweight or obesity: Evidence from South Africa | Azomahou et al. (2022) | Household | 2,711 households | South Africa | Undernutrition for children:   - Stunting: <-2SD height-for-age - Wasting: <-2SD weight-for-height - Underweight: <-2SD weight-for-age   Overweight for children   - Overweight: >+2SD height-for-age   Overnutrition for adults:   - Overweight/obese: BMI ≥25 kg/m²   Undernutrition for adults:   - Underweight: BMI <18.5 | DBM is defined as overweight mother and child having any form of undernutrition (stunting, wasting or underweight). |
|  | How does mode of delivery associate with double burden of malnutrition among mother-child dyads?: a trend analysis using Bangladesh demographic health surveys | Sutopa et al. (2022) | Household | 14,975 mother-child pairs | Bangladesh | - Stunting: <-2SD height-for-age - Wasting: <-2SD weight-for-height - Underweight: <-2SD weight-for-age   Overweight/obese: BMI ≥25 kg/m² | DBM is defined as overweight mother and child having any form of undernutrition (stunting, wasting or underweight). |
|  | Prevalence and associated factors of double and triple burden of malnutrition among child-mother pairs in Ethiopia: Spatial and survey regression analysis | Tarekegn et al. (2022) | Household | Total: 7,624 child-mother pairs | Ethiopia | - Stunting: <-2SD height-for-age - Wasting: <-2SD weight-for-height - Underweight: <-2SD weight-for-age - Overweight/obese: BMI ≥25 kg/m² | DBM is defined as overweight mother and child having any form of undernutrition (stunting, wasting or underweight). |
|  | Prevalence and socioeconomic determinants of the double burden of malnutrition in mother-child pairs in Latin America and the Caribbean | Otten et al. (2022) | Household | Total: 48,807 mother-child pairs | Latin America and the Caribbean (LAC) region: Bolivia, Colombia, Haiti, Honduras, Dominican Republic, Guyana, Guatemala, Peru | - Stunting: <-2SD height-for-age - Wasting: <-2SD weight-for-height - Underweight: <-2SD weight-for-age - Overweight/obese: BMI ≥25 kg/m² - Anemia: hemoglobin <12 g/dL for females - Anemia: hemoglobin <11 g/dL for children   Overweight for child: >+2SD height-for-age | DBM is defined as the following way:  overweight mother with at least one stunted child)  - overweight mother with at least one wasted child  - overweight mother with at least one anaemic child  - anaemic mother with at least one overweight child |
|  | The double burden of malnutrition and dietary patterns in rural Central Java, Indonesia | Lowe et al. (2021) | Household | Total: 1,521 households | Central Java, Indonesia | - Stunting: <-2SD height-for-age - Overweight/obese: BMI ≥25 kg/m² | DBM at the household level is typically defined as the presence of an overweight mother and a stunted child |
|  | Food insecurity and the double burden of malnutrition in Colombian rural households | Rosas et al. (2021) | Household | 2,350 mother-child pairs | Colombia | - Stunting: <-2SD height-for-age - Overweight/obese: BMI ≥25 kg/m² | DBM is the coexistence of a stunted child under 5 years of age and an overweight or obese mother |
|  | Predictor of Obese Mothers and Stunted Children in the Same Roof: A Population-Based Study in the Urban Poor Setting Indonesia | Berawi et al. (2021) | Household | Total: 428 households | Indonesia | - Stunting: <-2SD height-for-age - Overweight/obese: BMI ≥25 kg/m² | DBM in households, defined as the coexistence of an overweight/obese mother and a stunted child |
|  | Urbanization in Peru is inversely associated with double burden of malnutrition: Pooled analysis of 92,841 mother-child pairs | Daniel et al. (2021) | Household | 92,841 mother-child pairs | Peru | - Stunting: <-2SD height-for-age - Wasting: <-2SD weight-for-height - Underweight: <-2SD weight-for-age - Overweight/obese: BMI ≥25 kg/m² | DBM is defined as overweight mother and child having any form of undernutrition (stunting, wasting or underweight). |
|  | Trends and patterns of the double burden of malnutrition (DBM) in Peru: a pooled analysis of 129,159 mother-child dyads | Pomati et al. (2021) | Household | 129,159 mother-child dyads | Peru | - Stunting: <-2SD height-for-age - Wasting: <-2SD weight-for-height - Underweight: <-2SD weight-for-age - Overweight/obese: BMI ≥25 kg/m² | DBM is defined as overweight mother and child having any form of undernutrition (stunting, wasting or underweight). |
|  | Household-level double burden of malnutrition in Ethiopia: a comparison of Addis Ababa and the rural district of Kersa | Bliznashka et al. (2021) | Household | Total: 1454 households  - Addis Ababa: 592 households  - Kersa: 862 households | Ethiopia | - Stunting: <-2SD height-for-age - Overweight/obese: BMI ≥25 kg/m² | DBM in households, defined as the coexistence of an overweight/obese adult and a stunted child |
|  | Maternal height and double-burden of malnutrition households in Mexico: stunted children with overweight or obese mothers | Lucia et al. (2021) | Household | Total: 4706 mother-child dyads | Mexico (South, North, Centre, Mexico City) | - Stunting: <-2SD height-for-age - Overweight/obese: BMI ≥25 kg/m² | DBM is defined as the simultaneous presence of maternal overweight and child short stature. |
|  | Prevalence and regional variations of coexistence of child stunting and maternal overweight or obesity in Myanmar | Hong et al. (2020) | Household | 3954 mother-child pairs | Myanmar | - Stunting: <-2SD height-for-age - Overweight/obese: BMI ≥23 kg/m² | DBM is defined as a stunted child with an overweight mother living in the same household |
|  | Factors associated with double burden of malnutrition among mother-child pairs in India: A study based on National Family Health Survey 2015-16 | Patel et al. (2020) | Household | Total: 184,680 mother-child pairs | India | - Stunting: <-2SD height-for-age - Wasting: <-2SD weight-for-height - Underweight: <-2SD weight-for-age - Overweight/obese: BMI ≥25 kg/m² | DBM is defined as overweight mother and child having any form of undernutrition (stunting, wasting or underweight). |
|  | Prevalence of double burden on malnutrition at household level in four Latin America countries | Melendez et al. (2020) | Household | Total: 26,506 households  - Brazil: 2,664 mother-child pairs  - Bolivia: 5,363 mother-child pairs  - Colombia: 11,467 mother-child pairs  - Peru: 7,012 mother-child pairs | Brazil, Bolivia, Colombia, Peru | - Stunting: <-2SD height-for-age - Overweight/obese: BMI ≥25 kg/m² | DBM is defined as the simultaneous presence of maternal overweight and child short stature. |
|  | Patterns and determinants of the double burden of malnutrition at the household level in South and Southeast Asia | Biswas et al. (2020) | Household | Total: 798,961 households | South and Southeast Asia: Bangladesh, India, Nepal, Pakistan, Myanmar, Timor, Maldives, Cambodia | - Stunting: <-2SD height-for-age - Wasting: <-2SD weight-for-height - Underweight: <-2SD weight-for-age - Overweight/obese: BMI ≥25 kg/m² | DBM is defined as overweight mother and child having any form of undernutrition (stunting, wasting or underweight). |
|  | Double burden of malnutrition at household level: A comparative study among Bangladesh, Nepal, Pakistan, and Myanmar | Anik et al. (2019) | Household | Households: 18,459  - Bangladesh: 6,478  - Nepal: 2,670  - Pakistan: 5,770  - Myanmar: 3,541 | Bangladesh, Nepal, Pakistan, Myanmar | - Stunting: <-2SD height-for-age - Overweight/obese: BMI ≥25 kg/m² | DBM in households, defined as the coexistence of an overweight/obese adult and a stunted child |
|  | The double burden of malnutrition: an assessment of 'stunted child and overweight/obese mother (SCOWT) pairs' in Kerala households | Jayalakshmi et al. (2019) | Household | 344 mother and child pairs | Kerala, India | - Stunting: <-2SD height-for-age - Overweight/obese: BMI ≥25 kg/m² | DBM is defined as the simultaneous presence of maternal overweight and child short stature. |
|  | Prevalence and sociodemographic determinants of household level double burden of malnutrition in Bangladesh | Das et al. (2019) | Household | 5951 households | Bangladesh | - Stunting: <-2SD height-for-age - Wasting: <-2SD weight-for-height - Underweight: <-2SD weight-for-age - Overweight/obese: BMI ≥25 kg/m² | DBM is defined as overweight mother and child having any form of undernutrition (stunting, wasting or underweight). |
|  | Intra-household double burden of malnutrition in a North African nutrition transition context: magnitude and associated factors of child anaemia with mother excess adiposity | Sassi et al. (2019) | Household | 437 child-mother pairs | Middle East and North Africa region, specifically Tunisia | - Stunting: <-2SD height-for-age - Wasting: <-2SD weight-for-height - Underweight: <-2SD weight-for-age - Overweight/obese: BMI ≥25 kg/m² - Anemia: hemoglobin <12 g/dL for females - Anemia: hemoglobin <11 g/dL for children - Overweight for child: >+2SD height-for-age | DBM is defined as following way:  - Anaemic child and overweight mother  - Anaemic child and obese mother  - Overweight child and anaemic mother  - Stunted child and overweight mother  - Stunted child and obese mother  - Wasted child and overweight mother  - Wasted child and obese mother |
|  | Understanding the double burden of malnutrition in food insecure households in Brazil | Gubret et al. (2017) | Household | 4299 mother/child pairs | Brazil | - Stunting: <-2SD height-for-age - Overweight/obese: BMI ≥25 kg/m² | DBM is defined as maternal overweight and child stunting |
|  | Double-burden of malnutrition among the indigenous peoples (Orang Asli) of Peninsular Malaysia | Wong et al. (2015) | Household | Total: 1,285 households | Peninsular Malaysia | - Stunting: <-2SD height-for-age - Wasting: <-2SD weight-for-height - Underweight: <-2SD weight-for-age - Overweight/obese: BMI ≥25 kg/m² | DBM is defined as overweight mother and child having any form of undernutrition (stunting, wasting or underweight). |
|  | Prevalence and determinants of the dual burden of malnutrition at the household level in Puna and Quebrada of Humahuaca, Jujuy, Argentina | Prevalencia *et al.* (2014) | Household | 403 households | Argentina | - Stunting: <-2SD height-for-age - Overweight/obese: BMI ≥25 kg/m² | DBM at the household level refers to households with an overweight or obese mother and a stunted child. |
|  | Predictors of maternal and child double burden of malnutrition in rural Indonesia and Bangladesh 1-3 | Oddo et al. (2012) | Household | Total: 415,443 households  - Indonesia: 247,126 households  - Bangladesh: 168,317 households | Indonesia, Bangladesh | - Stunting: <-2SD height-for-age - Overweight/obese: BMI ≥25 kg/m² | DBM is defined as the simultaneous presence of maternal overweight and child short stature. |
|  | Socioeconomic disparities and the familial coexistence of child stunting and maternal overweight in guatemala | Lee et al. (2012) | Household | Total: 2492 households | Guatemala | - Stunting: <-2SD height-for-age - Overweight/obese: BMI ≥25 kg/m² | DBM is defined as coexistence of child stunting and maternal overweight |
|  | Peri-Urban, but Not Urban, Residence in Bolivia Is Associated with Higher Odds of Co-Occurrence of Overweight and Anemia among Young Children, and of Households with an Overweight Woman and Stunted Child | Jones et al. (2018) | Household & Individual | 3946 households | Bolivia | Undernutrition for children:   - Stunting: <-2SD height-for-age - Anemia: hemoglobin level <11.0 g/dl   Overweight for children   - Overweight: >+2SD height-for-age   Overnutrition for adults:   - Overweight/obese: BMI ≥25 kg/m²   Undernutrition for adults:   - Underweight: BMI <18.5 - Anemia: hemoglobin level <12 g/dL | DBM is defined as following way:  Individual level  - Concurrent overweight and anemia among women aged 15-49 years  - Concurrent overweight and anemia among children aged 6-59 months  - Concurrent overweight and stunting among children  Household level  - Households with an overweight woman and anemic child  - Households with an overweight woman and stunted child |
|  | Deforestation and Household-and Individual-Level Double Burden of Malnutrition in Sub-saharan Africa | Shankar et al. (2020) | Household & Individual | Total: 73,941 PSC  25,285 WCBA,  69,082 mother-child pairs | Sub-Saharan Africa: Benin, Cote d'Ivoire, Ghana, Guinea, Liberia, Mali, Nigeria, Senegal, Sierra Leone, Togo, Democratic Republic of Congo, Gabon, Zambia, Kenya, Rwanda | - Stunting: <-2SD height-for-age - Anemia: hemoglobin level <12.0 g/dl - Overweight/obese: BMI ≥25 kg/m² | Household-level: Co-occurrence of an overweight woman of childbearing age (WCBA) and a stunted pre-school child (PSC) within the same household.  Individual level: Co-occurrence of overweight and anemia within an individual WCBA.  Individual level: Co-occurrence of overweight and stunting within a PSC. |
|  | The dual burden of malnutrition in Colombia | Sarmiento et al. (2014) | Household & Individual | Total individuals: 160,696  - Children <5 years: 17,696  - School-aged children: 25,508  - Adolescents: 28,328  - Adults: 89,164  Total households: 10,487 | Colombia | - Among children 6–59 month, anemia was defined as hemoglobin <11 g/dL. For children 5–12 y old and female adolescents, anemia was defined as hemoglobin <12 g/dL. For male adolescents, anemia was defined as hemoglobin <13 g/dL, for adult women as hemoglobin <12 g/dL, and for pregnant women as hemoglobin <11 g/dL. - Stunting: <-2SD height-for-age - Overweight/obese: BMI ≥25 kg/m²   Stunting for child: >+2SD height-for-age | The dual burden of malnutrition was defined as the coexistence of overweight and stunting or anemia in the same person or household. |
|  | Various forms of double burden of malnutrition problems exist in rural Kenya | Fongar et al. (2019) | Household & Individual | Total individuals: 1058  - Female adults: 558  - Male adults: 316  - Children: 184  Total households: 835 | Nyanza region, Western Kenya (Kisii and Nyamira Counties) | - Overweight/obese for adult: BMI ≥25 kg/m²   For child under nutrition:   - Stunting: <-2SD height-for-age - Wasting: <-2SD weight-for-height - Underweight: <-2SD weight-for-age - body-mass-index-for-age Z-scores (BAZ)<-2 SD   Micronutrient deficiency:  To identify micronutrient deficiency, estimated average requirements (EAR) were used for each nutrient, taking individual gender and age into account. EAR thresholds used to define micronutrient deficiency. EAR thresholds values are available in the “Additional file 2” of the original article.  Note that, it is a very large table, so, we are not able to show here. | DBM is defined as different way:  - DB 1: Adult overweight/obese and micronutrient-deficient  - DB 2: Child overweight (BAZ > + 2 SD) and micronutrient-deficient  - DB 2.1: Child overweight (WHZ > + 2 SD) and micronutrient-deficient  - DB 3: Child overweight (BAZ > + 2 SD) and stunted (HAZ < -2 SD)  - DB 3.1: Child overweight (WHZ > + 2 SD) and stunted (HAZ < -2 SD)  - DB 4: Adult overweight/obese and child underweight (BAZ < -2 SD)  - DB 5: Adult overweight/obese and child underweight (WAZ < -2 SD)  - DB 6: Adult overweight/obese and child stunted (HAZ < -2 SD)  - DB 7: Adult overweight/obese and child wasted (WHZ < -2 SD)  - DB 8: Adult overweight/obese and child micronutrient-deficient |
|  | The double burden of malnutrition in indigenous and nonindigenous Guatemalan populations 1-4 | Manuet et al. (2014) | Household & Individual | Households: 22,990 (2008)  - Women of Reproductive Age: 16,819 (2008)  - Children aged 0-59 months: 10,775 (2008) | Guatemala | Undernutrition and overnutrition for women:   - Underweight: <18.5 - Overweight/obese: BMI ≥25 kg/m² - Short stature: height <145 cm - Anemia: hemoglobin <12 g/dL   Undernutrition for children   - Stunting: <-2SD height-for-age | - Household level DBM (stunted child and overweight mother)  - Individual level DBM (stunting/short stature and overweight or anemia and overweight) |
|  | The Nutritional Dual-Burden in Developing Countries -How is it Assessed and What Are the Health Implications? | Silva et al. (2012) | Household & Individual | Total: 116 (58 mother-child pairs) | Mexico | These articles used anthropometric indicators and nutritional status cut-off points based on guidelines from the WHO, CDC, and the International Obesity Task Force (IOTF). Detailed measurements are provided within the articles.  Please note that due to the large size of the table, it is not included here. | DBM is defined as the coexistence of stunting and overweight/obesity in individuals or within mother-child pairs. |
|  | Double malnutrition and associated factors in a middle-aged and older, rural South African population | Seedat et al. (2024) | Individual | Total: 250 | South Africa | - Overweight: BMI ≥25.0 kg/m² - Iodine deficiency: a median UIC of <100 μg/L - Anemia: hemoglobin <13 g/dL for males or <12 g/dL for females | Individual level DBM is defined as:  - Overweight/obesity and anaemia  - Overweight/obesity and iodine insufficiency  - Overweight/obesity and any micronutrient deficiency (anaemia and/or iodine insufficiency) |
|  | The Double Burden of Malnutrition is Associated with Continued Breastfeeding and Early Consumption of Ultra-Processed Drinks in Socially Vulnerable Brazilian Children | Gonzaga et al.(2024) | Individual | Total: 561 | Northeast Brazil | - Short stature: <-2SD height-for-age - Overweight: >+2SD height-for-age | DBM at individual level is the coexistence of short stature and overweight in the same child. |
|  | Prevalence of the Double Burden of Malnutrition among Adolescents: Associations with Lifestyle Behaviors and Clusters of Social Determinants | Manuet el al. (2024) | Individual | Total: 1152 | Brazil | BMI-z-score: The adopted cutoff points were as follows: Z-score < −3 (very low weight); Z-score ≥ −3 and <−2 (low weight); Z-score ≥ −2 and ≤1 (normal weight); Z-score > 1 and ≤2 (overweight); and Z-score > 2 (obesity)   - Vitamin D deficiency or hypovitaminosis is characterized by 25(OH)D levels below 20 ng/mL, while vitamin D insufficiency is defined by 25(OH)D levels | DBM is defined as the coexistence of obesity and vitamin D deficiency |
|  | Double burden of malnutrition among women of reproductive age: Trends and determinants over the last 15 years in India | Prithishkumar et al. (2024) | Individual | NFHS-3: 117,956; NFHS-4: 668,563; NFHS-5: 696,990 | India | - Anemia: hemoglobin <12 g/dL for females - Overweight/obese: BMI ≥25 kg/m² | Presence of both (i) underweight and anaemia, and (ii) overweight/ obesity and anaemia, within the same individual group was categorised as having DBM. |
|  | Double burden of malnutrition in Afghanistan: Secondary analysis of a national survey | Fahim et al. (2023) | Individual | Total: 126,890 individuals | Afghanistan | - Stunting: <3rd percentile of height-for-age based on WHO criteria in 0-19y - Wasting: <3rd percentile (WHO cut-off points) age- and sex-specific weight-for-length for children aged <2 y and <3rd percentile (WHO cut-off points) age- and sex-specific BMI-for-age for children and adolescents aged 2-19y - Underweight: <3rd percentile (WHO cut-off points) age- and sex-specific weight-for-age for children aged 0-10y, <3rd percentile (WHO cut-off points) age- and sex-specific BMI-for-age for adolescents aged >10–19 y and BMI<18.5 kg/m2 for those aged >19 y - 4) Vitamin D deficiency: <20ng/ml - 5) Anemia: hemoglobin concentrations of less than 11 g/dL among children aged <5.0 years, and less than 11.5 g/dL among those aged ≥5.0, <12.0 years. These cut-off points was less than 12.0 g/dL among those aged≥12.0 and <15.0 years. In non-pregnant females aged ≥ 15.0 years, this cut-off point was 12.0 g/dL - Vitamin A deficiency: serum retinol concentrations below a cut-off of 0.70 μmol/L (or 20 μg/dL) - Iodine deficiency: a median UIC of <100 μg/L - Overweight/obese was defined as >85th percentile (WHO cut-off points) age- and sex-specific weight-for-length for children aged <2 y and >85th percentile (WHO cut-off points) age- and sex-specific BMI-for-age for those aged 2–19 y and BMI ≥25 kg/m2 for those aged >19 y | Individual DBM was defined as the co-existence of "overweight" along with "stunting or micronutrient deficiencies" (including anemia, vitamin A deficiency, vitamin D deficiency and iodine deficiency) |
|  | Dual burden of individual malnutrition in children 1-4 years: Findings from the Colombian nutritional health survey ENSIN 2015 | Castillo et al. (2023) | Individual | Total: 6807 | Colombia (Atlantic, Central, Pacific, Eastern, Bogotá) | - Iron deficiency was defined as serum ferritin values below 12 μg/L - Zinc deficiency by serum zinc values below 65 μg/L - Vitamin A deficiency by serum retinol values below 20 μg/dL - Vitamin D deficiency by serum 25-OH-D values below a concentration of 30 nanomoles per litre (nmol/L) - Stunting: <-2SD height-for-age   Overweight for child: >+2SD height-for-age | DBM is defined by the presence of overweight and stunting and/or micronutrient deficiency (vitamin A, D, zinc, or iron) |
|  | Trends in the Intraindividual Double Burden of Overweight/Obesity and Anemia among Adult Women Living in 33 Low-and Middle-Income Countries: A Secondary Analysis of Demographic and Health Surveys from 2000-2019 | Irache *et al.* (2023) | Individual | 1,648,308 nonpregnant adult women | African region, Eastern Mediterranean region, European region, Americas region, Southeast Asian region, Western Pacific region | - Overweight/obese: BMI ≥25 kg/m² - Anemia: hemoglobin concentrations <12.0 g/dL | DBM is defined as the coexistence of overweight or obesity and anemia |
|  | The double burden of overweight or obesity and anemia among women married as children in India: A case of the Simpson's paradox | Datta et al. (2022) | Individual | Total: 473,052 | India | - Anemia: hemoglobin level <12.0 g/dl - Overweight/obese: BMI ≥25 kg/m² | DBM is defined as the coexistence of overweight or obesity and anemia. |
|  | Sex Differences in Dietary Patterns of Adults and Their Associations with the Double Burden of Malnutrition: A Population-Based National Survey in the Philippines | Kaliora et al. (2022) | Individual | Total: 8957  - Male: 4465  - Female: 4492 | Philippines | - Anemia: hemoglobin <13 g/dL for males or <12 g/dL for females - Vitamin A deficiency: serum retinol of <10 μg/dL indicated - Hyperglycemia was characterized as a fasting blood glucose ≥110 mg/dL - dyslipidemia was characterized as having an HDL cholesterol <40 mg/dL for males or <50 mg/dL for females, or triglyceride ≥150 mg/dL - Hypertension was denoted by a blood pressure measurement of ≥140/≥90 mmHg - Overweight/obese: BMI ≥25 kg/m² - Waist circumference was obtained with a calibrated tape measure midway between the lowest rib and tip of the hip bone while the participant was standing and breathing normally, and expressed to the nearest 0.1 cm. Abdominal obesity was defined as waist circumference ≥102 cm for males or ≥88 cm for females - A blood pressure measurement of ≥140/≥90 mmHg indicated hypertension | DBM is defined as the co-existence of underweight or anemia or vitamin A deficiency or iodine insufficiency and at least one cardiometabolic risk factor. |
|  | Prevalence and Determinants of the Co-Occurrence of Overweight or Obesity and Micronutrient Deficiencies among Adults in the Philippines: Results from a National Representative Survey | Juras et al. (2021) | Individual | Total: 17,010  - Male: 8,536  - Female: 8,474 | Philippines | - Overweight/obese: BMI ≥25 kg/m² - Anemia: hemoglobin < 13 g/dL for males and <12 g/dL for females - vitamin A deficiency: serum retinol with a cut-off of <10 μg/dL | Individual level DBM is defined as:  - Overweight/obesity and anemia  - Overweight/obesity and anemia or vitamin A deficiency  - Overweight/obesity and anemia or vitamin A deficiency or iodine insufficiency |
|  | Intraindividual Double Burden of Malnutrition in Chinese Children and Adolescents Aged 6-17 Years: Evidence from the China Health and Nutrition Survey 2015 | Xiao Hu et al. (2021) | Individual | Total: 1555  - Boys: 806  - Girls: 749 | China | Children and adolescents’ obesity were defined as BAZ > +2 SD, overweight was defined as (BAZ > +1 SD), and underweight was defined as BAZ < −2 SD.   - Micronutrient Deficiency: Cut-offs are not clearly defined. Just mention that “We applied estimated average requirement (EAR) as the cut-offs to assess dietary intake of the micronutrients in present study.” However, not provide any references for EAR values. | Individual DBM is defined as the coexistence of overweight/obesity and dietary micronutrient intake insufficiency. |
|  | The Double Burden of Malnutrition at the Individual Level Among Adults: A Nationwide Survey in the Philippines | Susan et al. (2021) | Individual | Total: 17,157  - Male: 8,596  - Female: 8,561 | Philippines | - Anemia: hemoglobin <13 g/dL for males or <12 g/dL for females - Vitamin A deficiency: serum retinol of <10 μg/dL indicated - Iodine insufficiency:  The acid digestion/colorimetric method was employed to evaluate the UIE levels with a cut-off of <50 μg/dL - Overweight/obese: BMI ≥25 kg/m² - Waist circumference was obtained with a calibrated tape measure midway between the lowest rib and tip of the hip bone while the participant was standing and breathing normally, and expressed to the nearest 0.1 cm. Abdominal obesity was defined as waist circumference ≥102 cm for males or ≥88 cm for females - A blood pressure measurement of ≥140/≥90 mmHg indicated hypertension - Hyperglycemia was defined as fasting glucose ≥110 mg/dL; low HDL cholesterol as <40 mg/dL for males and <50 mg/dL for females; and hypertriglyceridemia as triglycerides ≥150 mg/dL. Participants were classified as having cardiometabolic risk factors (CMRF) if they had any of the following: overweight/obesity or abdominal obesity, hypertension, hyperglycemia, or dyslipidemia (low HDL or high triglycerides) | DBM at individual level is defined as following way:  - Co-occurrence of underweight and at least one cardiometabolic risk factor (Uw + ≥1 CMRF)  - Co-occurrence of anemia and at least one cardiometabolic risk factor (An + ≥1 CMRF)  - Co-occurrence of vitamin A deficiency or iodine insufficiency and at least one cardiometabolic risk factor (Other MND + ≥1 CMRF)  CMRF measurements included the following: overweight/obesity or abdominal obesity, hypertension, hyperglycemia, and dyslipidemia (low HDL cholesterol or hypertriacylglycerolemia) |
|  | Intra-Individual Double Burden of Malnutrition among Adults in China: Evidence from the China Health and Nutrition Survey 2015 | Huang et al. (2020) | Individual | Total: 6602  - Men: 3699  - Women: 2903 | China | - Overweight/obese: BMI ≥25 kg/m² - Micronutrient deficiency   Using the China Food Composition Table, the authors calculated the average three-day intake of:   - Total energy - Micronutrients (e.g., vitamins and minerals) - Retinol intake was estimated using a conversion formula: μgRAE = μg retinol + μg carotenes/12 | DBM is defined as the coexistence of overweight/obesity and micronutrient deficiency. |
|  | The prevalence and correlates of the double burden of malnutrition among women in Ghana | Kushitor et al. (2020) | Individual | Total: 4,337 women | Ghana | - Anemia: hemoglobin <12 g/dL for females - Overweight/obese: BMI ≥25 kg/m² | Women who suffered simultaneously from underweight or overweight/obesity and anaemia were considered as having the DBM. |
|  | National income and macro-economic correlates of the double burden of malnutrition: an ecological study of adult populations in 188 countries over 42 years | Talukdar et al. (2023) | Population | Not mentioned | North America and Europe, East Asia, South Asia, Latin America, Middle East and North Africa, Sub-Saharan Africa | - Overweight: BMI ≥25.0 kg/m² - Underweight: BMI <18.5 kg/m² | A country was considered to have the DBM in a year when adult overweight and underweight prevalence was each 10% or more in that year. |
|  | The double burden of malnutrition in under-five children at national and individual levels: observed and expected prevalence in ninety-three low-and middle-income countries | Lerm et al. (2020) | Population | Total: 825,633 children | West and Central Africa, Eastern and Southern Africa, Middle East and North Africa, Europe and Central Asia, South Asia, East Asia and the Pacific, Latin America and the Caribbean | - Stunting: <-2SD height-for-age - Overweight/obese: BMI ≥25 kg/m² | At the population level, DBM is measured by the prevalence of stunting and overweight, using thresholds of 20% for stunting and 10% for overweight. |
|  | Dynamics of the double burden of malnutrition and the changing nutrition reality | Popkin *et al.* (2019) | Population | Not mentioned | sub-Saharan Africa, south Asia, east Asia, Pacific, Latin America and the Caribbean, eastern Europe, central Asia | In low- and middle-income countries (LMICs), DBM is identified when there is a prevalence of:   - Wasting > 15% - Stunting > 30% - Thinness in women > 20% - Overweight in children or adults > 20%   The specific cutoffs include:   - Wasting: Weight-for-height Z score < -2 - Stunting: Height-for-age Z score < -2 (for children aged 0-4 years) - Thinness in women: BMI < 18.5 kg/m² - Overweight: BMI Z score > 2 in children; BMI > 25 kg/m² in adults. | DBM at the country level is defined by the coexistence of high levels of both undernutrition and overweight/obesity within a population. |
